# COVID-19 and the Twin Transition: How the Recovery Can Boost Sustainable and Inclusive Growth

**DOI:** 10.1007/s10272-020-0931-z

**Published:** 2020-12-01

**Authors:** Debora Revoltella

**Affiliations:** Volkswirtschaftliche Analysen, European Investment Bank, 98-100, Boulevard Konrad Adenauer, L-2950 Luxembourg, Luxembourg

## Abstract

Europe has a window of opportunity to embark on a recovery that tilts towards digitalisation and a greener economy, and stands to generate sustainable benefits for its people.

The COVID-19 pandemic struck the EU economy hard. Economic activity fell dramatically in the first half of the year in the EU. Optimism for a quick turnaround in the third quarter of 2020 was replaced by renewed fears about the spread of the virus, and new lockdown measures were gradually put in place in autumn. Still in the mist of uncertainty, the EU economy is expected to return to growth at a slow pace.

Uncertainty continues to impede forceful recovery. Uncertainty linked to the pandemic has three dimensions:

- medical – related to the timing for an effective vaccine development and deployment, successes in immunisation, etc.
- economic – second-round effects of the economic crisis, e.g. persistent shift in consumers' attitude towards precautionary savings, potential round of delayed corporate bankruptcies or unemployment
- long-term implications – linked to possible structural changes induced by COVID-19, for example, changes in preferences, rethinking of the global value chains, emergence of new business and organisational models, as well as stronger role of the state in economic terms.

High levels of uncertainty pose major concerns to firms, leading many to postpone or cancel investment. Findings based on the latest European Investment Bank investment survey (EIB, 2020) show that uncertainty remains a key impediment to investment, cited as a constraint by 81% of EU firms. The coronavirus pandemic has strongly

Figure 1

**COVID-19 impact on investment in the short term**

Share of firms

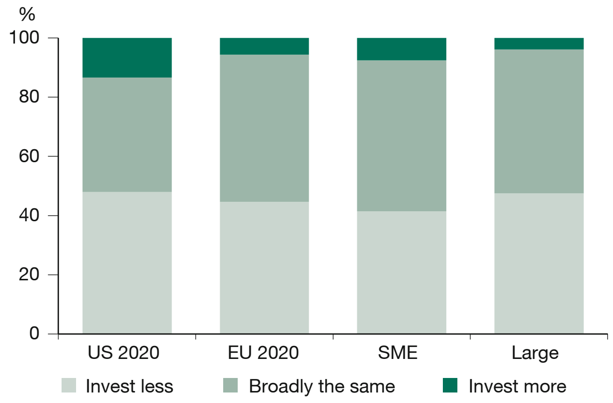

Note: Base: All firms who have invested in the last financial year (excluding do not know/refused responses). Q: What proportion of total investment was for (a) replacing existing buildings, machinery, equipment, IT; (b) expanding capacity for existing products/services; (c) developing or introducing new products, processes, services?

Source: IPSOS, 2020.

Figure 2

**COVID-19 long-term impact points to a 'new normal'**

Share of firms claiming COVID-19 will impact each factor

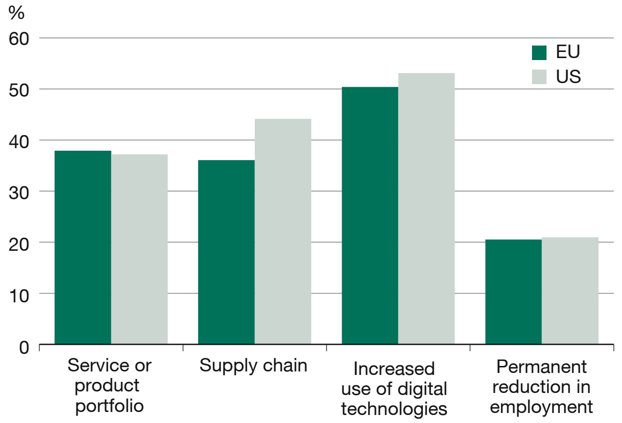

Note: Base: All firms who have invested in the last financial year (excluding do not know/refused responses). Q: What proportion of total investment was for (a) replacing existing buildings, machinery, equipment, IT; (b) expanding capacity for existing products/services; (c) developing or introducing new products, processes, services?

Source: IPSOS, 2020.

affected corporate investment across the EU. Almost half (45%) of firms intend to invest less in the current financial year due to the coronavirus (Figure 1). Among those that have investment plans in the current financial year, around one-third (35%) of firms say they will delay or abandon at least some of these plans due to COVID-19. Around one-fifth (18%) expect to continue with their investment plans on a reduced scale. Uncertainty about the economic environment, leading to a 'wait-and-see' approach, is compounded by a more challenging financial situation for corporations. Notwithstanding exceptional policy support, closures or reduced activity have given rise to cumulative revenue losses ranging from 2%-3.5% of assets for large corporations up to 6%-10% for SMEs in the last six months. To re-absorb those losses at least partially, firms will have to review their future plans. With internal finance strongly constrained, a trade-off emerges between an attempt to preserve, at least in part, investment and mounting corporate leverage.

## The 'new normal'

A short-term drop in investment collides with enhanced long-term needs in order to adapt to a COVID-19 induced 'new normal'. The EU economy will not revert to the 'old normal' in the aftermath of the pandemic. Digitalisation has received a boost over the last few months and is widely expected to shape the 'new normal' (Figure 2).

## Increasing digitisation

Firms across the EU need to step up the pace to maintain competitiveness in the post-pandemic environment shaped by increasing digitalisation. Almost four out of ten firms (37%) are still non-digital, i.e. have not adopted key digital technologies (EIB, 2019; EIB, 2020). Reasons for their slow adaptation include a lack of awareness about technologies available, their potential benefits and the urgency of the situation. Moreover, there are issues in firms' operating environments that slow technology diffusion. Some 16% of EU firms find the (lack of) digital infrastructure to be a major impediment to digitalisation, i.e. three times more than in the US (5%). Innovation and revisiting the structure of the global value chains are other areas of potential structural change post-COVID-19.

## Climate change is here to stay

The EU Commission and the EU Parliament have reconfirmed the ambitious EU decarbonisation targets, requiring a prompt transformation of the EU economy. The majority of EU firms (58%) report that they already feel it has an impact on their business. With two-thirds reporting that they already invest in climate change or plan to do so, EU firms show greater responsiveness to the climate challenge than US peers (46%). Uncertainty about regulation and taxation, however, are key factors holding firms back to step up activities to tackle climate change and the risks related to it.

## Labour market impact

The digital and the green transition are going to impact on EU labour markets. The transition's effect will vary widely, depending on geography and labour market groups. Recent advances in digital technologies have tended to benefit high-skilled workers and those in less-routine occupations. At the same time, it has been associated with growing spatial disparities, with people and businesses increasingly clustering in favoured urban locations to live and work. The greening of EU economies needed to meet the EU's goal of neutral carbon emissions will require major industrial transformation. Carbon-intensive activities are more prone to job loss during the green transition with risks spatially concentrated. Change caused by structural transformation and the risks to employment that come with it will have to be dealt with against the backdrop of a less favourable labour market situation in the immediate aftermath of the pandemic. In a worst-case scenario, post-pandemic labour markets could see an increase in skill mismatches that coincide with higher unemployment.

Labour market risks from both forces of structural change coincide in some places. Where job loss caused by automation and greening coincide, alternate employment will be harder to find, all else being equal. Analysing twin transition risks, we find that high double exposures to both types of structural change cluster in Central and Eastern Europe.

Regions with high exposure to twin risks tend to be poorer, less densely populated and often have labour markets that include some structural difficulties. Comparing the regions with high exposure to both risks to the regions facing lower risks suggests that higher-risk regions are already facing more challenges. None of the regions with the highest twin transition risks are among the 30 most R&D-intensive regions in the EU. Accordingly, firms in regions with high twin transition risks tend to invest less in intangibles.

Divergences in regional performance will be more difficult to address in the coming years. European economies will continue to reel from the coronavirus crisis, and (structural) unemployment could remain an issue. This applies to regions that stand to be directly affected by transition risks and to individual countries. In this respect, parts of Southern Europe face a more challenging situation as local labour markets have not yet fully recovered from the financial and sovereign debt crisis and have experienced persistent structural difficulties.

Digitalisation and greening can be a source of job creation, with policies needed to provide a boost. Structural changes come with job destruction and job creation effects. Jobs to emerge from greening are expected for instance in renewable energy, recycling or construction. In the longer term, the green transition is forecast to be job neutral or even add to employment in the EU. For digitalisation, new technologies have not yet led to job destruction on balance. EIB investment survey results further corroborate employment creation effects for digital firms. Digital firms have been more likely to add jobs than their non-digital peers over the past three years. Gereben and Wruuck (2020) find job creation effects by digital firms across the EU and particularly in Southern Europe.

## Keys to the twin transition

The twin transition requires skill sets to adapt. Jobs created are not the same as the ones lost, and structural shifts come with changes in the demanded skill sets. For example, shifts linked to digitalisation have become apparent in recent years, with high-skilled jobs increasing and firms, particularly innovative ones seeking more advanced or new skill sets, struggling to find the right people (see EIB, 2018, 2019). Similarly, the greening of the EU economy will change skills needed in a number of occupations, e.g. architects or engineers well versed in building renovations or green technologies will be increasingly sought after.

Learning is key to make structural transformations a success. Changes can raise the risk of skill mismatches and shortages if skill supplies react (too) slowly. Supporting learning in times of structural transformation, including reskilling and upskilling measures, is key to mitigate the risk of exclusion from the labour market, mitigate inequalities and allow people to seize opportunities related to changes.

Digital technologies and education helped create resilience to the COVID-19 shock. The crisis also underscored how education and digitalisation can be a source of resilience. Individuals' concerns about unemployment were lower in European regions where the use of digital technologies is more prevalent and where a smaller share of the population had lower levels of education (Figure 3).

Managing the fallout from the pandemic, while at the same time building a more digital and green economy, requires strong policy support. The rebound of the EU economy will take time but we have a chance to rebuild it for the better. Moving into the 'new normal', digitalisation

Figure 3
**Regional impact of the COVID-19 shock on unemployment concerns**

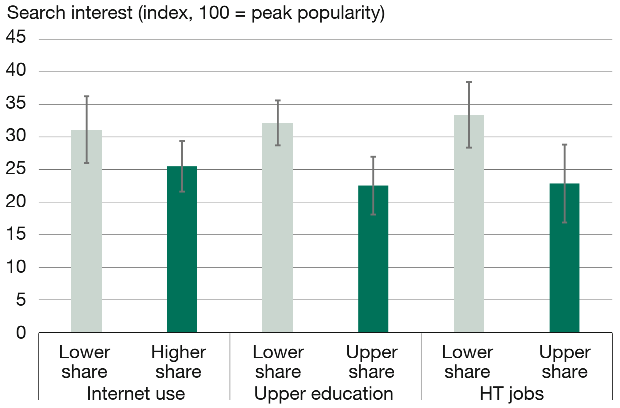

Notes: Predicted Google search intensity for the unemployment topic for an average region facing 1000 newly identified COVID-19 cases. Lower shares denotes 25th and upper shares denotes 75th percentiles of variable regional distributions. Internet use reflects share of population which use internet on daily basis, upper education corresponds to population share with education level 3 or above, and HT jobs denotes the employment share in science and technology sectors. Based on 306 EU regions, excluding capital regions. Daily observations between January and May 2020, starting from cumulative 100 cases in a country. The model controls for regions disposable income and population and includes fixed effects.

Source: EIB Econ calculations, 2020.

and the green transition can be a source of job creation. For this to happen, policymakers need to set the right incentives, provide support through investment and labour market policies to help preserve employment rather than specific jobs, and facilitate job transitions.

Climate action has the potential to boost employment in the short to medium term, and in turn, support a job-rich recovery. Employment opportunities linked to greening can be a source of local job growth, offering 'meaningful' transition opportunities, for example from mining to renewables. Moreover, these new jobs can help to sustain employment, support demand and strengthen the recovery from the coronavirus pandemic. For green jobs to materialise, Europe needs to invest, particularly in skills, and create the right mix of incentives.

A strong skill base can support job creation from digitalisation. Digitalisation can be more labour-saving or labour augmenting. Which technology firms adapt and the quality and the quantity of jobs they (expect to) create depend on industry structures and the business environment, including the short-term outlook and longer-term obstacles. Adopting more jobs suited to technologies is more likely where the availability of skill sets to support this is better. This includes expert digital skills but also more basic user skills, which support incentives for technology adoption, digital innovation and the creation of new business models. At the same time, diffusion of digital skill sets is crucial to avoid further polarisation on labour markets and deepening of digital divides in society.

## The long game

Europe has a window of opportunity to embark on a recovery that tilts towards digitalisation and a greener economy, and stands to generate sustainable benefits for its people. Failure to seize the opportunity to turn the European economy around will carry a high cost. Megatrends are here to stay, and new technologies are key to providing environmental and social sustainability. A failure to act now would mean a slower and less sustainable recovery for the next years. In the longer term, it would mean a less healthy planet, a further loss of competitiveness and economic significance for Europe in the decades to come, and a high risk of exacerbating existing inequalities while new ones emerge.

A mix of immediate and longer-term support is needed to avoid deepening inequalities and to facilitate adaptation to structural change in the current situation. For a job-rich recovery powered by digitalisation and greening, short-term actions need to align with long-term strategy. Moreover, efforts need to be well coordinated across Europe, and private and public sector incentives and actions need to align. This requires clarity in terms of targets and policy guidance, particularly for climate change. It also calls for stepping up action to fully realise the digital single market and support the broadening of digital skills. Finally, a strong focus on promoting skills can help people to transition to more promising areas of economic activity in the short to medium term, mitigating risks of scarring effects and long-term unemployment. In the longer term, reforms to adult education coupled with investment in education quality form the basis to boost innovation and advance the digital and green transition in a human-centric way. Investment in each of the three areas will complement the others and raise the economic and societal returns.
